# Supplementary material for: Action repetition biases choice in context-dependent decision-making
Source: Commun Psychol. 2025 Nov 26;3:177. doi: 10.1038/s44271-025-00363-x (PMC12660811; doi:10.1038/s44271-025-00363-x)
Supplement: Supplementary file 2 — Supplementary Information [file 44271_2025_363_MOESM2_ESM.pdf]

Supplementary Information

Supplementary Table 1 Model comparison overview. For tasks p1 to p4.2 relative learning and other normalization procedures do not differ substantially in their predictions. Thus, for these tasks relative value learning is the only normalization model. For tasks with different reward magnitudes and/or more than two options per context we instead fitted divisive- and range normalization models.

Model	Dataset (task)				
	p1.1, p1.2, p1.3, p2, p3, p4.1, p4.2	g1, g2	Exp2, Exp 3 (Klein et al. 2017)	Exp1, Exp2 Bavard et al. 2018	Exp3a, Exp3b (Bavard and Palminteri 2023)
REP (standard Q-learning + repetition bias)	x	x	x	x	x
ABS (standard Q-learning)	x	x	x	x	x
IER (intrinsic enhanced reward[1])	x	x	x	x	x
REL (relative value-learning)[2]	x	-	x	-	-
DIV (divisive normalization)[3–5]	-	x	x	x	x
RANGE (range normalization)[6]	-	x	x	x	x
RANGE$\boldsymbol{\omega}$ (range normalization + $\boldsymbol{\omega}$ exponent)[6]	-	x	x	x	x

Supplementary Table 2 Correlation coefficients for relative choice-frequency correlation via *brms* between learning and transfer phase for all options pairs encountered during transfer. For tasks p1, p2, p3, p4.1, p4.2 and Klein et al. 2017[2] Exp. 2 and 3 (first row) and g1, g2 (second row) data was pooled across tasks.

Pearson r [CI]	Spearman ρ [CI]	BF_{10} Pearson/ Spearman	n	Task	Shapiro-Wilk (learning)	Shapiro-Wilk (transfer)
0.79 [0.53, 0.93]	0.79 [0.54, 0.93]	671/ 750	16	p1-p4.2, Klein et al. 2017 exp 2 and 3	1.32e-01	1.09e-01
0.92 [0.82, 0.97]	0.97 [0.94, 0.99]	>1000/ >1000	18	g1-g2	5.94e-02	2.81e-03*
0.68 [0.44, 0.85]	0.69 [0.46, 0.85]	>1000/ >1000	28	Bavard et al. (2018) Exp. 1	3.06e-01	1.16e-01
0.89 [0.79, 0.95]	0.81 [0.64, 0.91]	>1000/ >1000	28	Bavard et al. (2018) Exp. 2	8.40e-03*	5.37e-02
0.92 [0.88, 0.95]	0.91 [0.86, 0.95]	>1000/ >1000	66	Bavard and Palminteri (2023) Exp. 3a	1.70e-04*	1.36e-05*
0.90 [0.84, 0.94]	0.86 [0.78, 0.91]	>1000/ >1000	66	Bavard and Palminteri (2023) Exp. 3b	8.76e-05*	5.60e-05*
0.88 [0.84, 0.90]	0.87 [0.84, 0.90]	>1000/ >1000	222	All datasets	1.50e-05*	6.26e-09*
0.87 [0.77, 0.93]	0.85 [0.74, 0.92]	>1000/ >1000	40	loss preference from Bavard et al. 2018	1.36e-02	3.40e-01
0.56 [0.23, 0.79]	0.62 [0.32, 0.82]	31/109	25	equal absolute value (pooled across datasets) 14 points bandits from Bavard and Palminteri (2023) excluded due to low choice rate)	2.09e-03*	1.54e-03*
0.56 [0.19, 0.82]	0.61 [0.25, 0.84]	49/23	18	Equal relative values (pooled across datasets) 14 points bandits from Bavard and Palminteri (2023) excluded due to low choice rate)	0.048*	0.067

Supplementary Table 3 Quantitative model comparison via the DIC [7] for all models with separate learning rates for chosen and unchosen options without a learning decay mechanism. For tasks p1-p4.2 and the two datasets from Klein et al. 2017 we compared ABS, REL, REP and IER models. For tasks g1, g2 and the datasets from Bavard et al. (2018) and Bavard and Palminteri (2023) we compared ABS, DIV, RAN, RAN$\boldsymbol{\omega}$ and IER models. We further fitted each model twice using two different learning rules. For results with context specific learning rates that decay over time (given how often a context was observed) see Supplementary Table 10 and 11 correspond to model versions with context-specific decaying learning rates (see methods for details). Further, we fitted two additional version of the REP model, where the repetition bias mechanism was only applied to the learning phase (second entry in the REP column) and one version for the two datasets from Bavard and Palminteri (2023) where the REPbias was not applied to the second-best stimulus in blocked trials [values in squared brackets].

**Dataset (feedback)**	**DIC(ABS)**	**DIC(REL)**	**DIC(DIV)**	**DIC(RAN) f**	**DIC(RANω)**	**DIC(REP)**	**DIC(IER)**
**Task p1 (full)**	4584.21	4633.33	-	-	-	4215.74 (4218.43)	4580.97
**Task p1.1**	1759.79	1811.29	-	-	-	1627.91	1759.76
**Task p1.2**	1436.62	1407.50	-	-	-	1329.39	1435.32
**Task p1.3**	1319.20	1299.91	-	-	-	1201.70	1318.86
**Task p2 (partial)**	2846.81	2844.55	-	-	-	(2697.38)	2840.97
**Task p3 (full)**	3771.47	3957.94	-	-	-	3493.84 (3457.40)	3772.71
**Task p4.1 (full)**	1739.12	1782.94	-	-	-	1589.33 (1650.76)	1736.45
**Task p4.2 (full)**	1603.67	1590.14	-	-	-	1401.43 (1389.76)	1603.46
**Task g1 (full)**	2305.55	-	3030.03	3036.28	2386.59	2114.407 (2349.3)	2047.44
**Task g2 (partial)**	4243.97	-	5702.21	4615.32	4506.58	4292.12 (4359.31)	4230.01
**Klein et al. 2017 Exp2 (full)**	1803.455	1819.59	-	-	-	1506.33 (1534.29)	1806.93
**Klein et al. 2017 Exp3 (full)**	1549.86	1701.26	-	-	-	1436.74 (1437.22)	1536.3
**Bavard et al. 2018 Exp1 (mixed)**	6423.7	-	8977.5	6634.8	6642.5	4797.0 (4987.82)	5990.3
**Bavard et al. 2018 Exp2 (full)**	12384.5	-	15055.6	11605.1	11605.6	9754.4 (9537.31)	11120.7
**Bavard et al. 2023 Exp3a (full)**	30593.2		30537.3	28041.48	28061.1	27429.9 [22063.8] (22212.27)	27785.4

**Bavard et al. 2023 Exp3b (full)**	32065.4		33015.5	30201.82	29432.8	29374.52 [24316.81] (25612.4)	29768.2

Supplementary Table 4 Complete pseudorandomized reward sequences for all probabilistic tasks p1.1 to p4.2. Sequences were randomly constructed (except for manual changes at the beginning of task p4.1 and p4.2) with constrains that true reward probabilities should be evident around every 10 to 12 trials and that the last 4 to 5 last trials for the best options in each context are equally rewarded (except for task p1.2).

**Task**	**LC/LG Context sequence (reward = 1; no reward = 0)**	**HC/HG sequence (reward = 1; no reward = 0)**
**p1.1**	A_{LC} (0.7):<br>0 1 1 1 1 1 0 1 0 1 1 0 1 1 1 0 1 1 1 0 0 0 1 1 1 1 1 0 1 1<br>B_{LC} (0.5):<br>1 0 0 1 1 1 1 0 1 1 1 0 0 1 1 0 0 0 1 1 0 0 1 1 0 0 0 1 0 0	C_{HC}(0.7):<br>1 0 1 1 1 0 1 1 1 1 1 1 1 0 1 0 0 0 1 1 0 1 1 0 1 1 1 0 1 1<br>D_{HC}(0.2):<br>1 0 0 0 0 0 0 1 0 1 0 0 0 0 0 0 0 0 1 1 0 0 0 0 0 0 1 0 0 0
**p1.2**	A_{LC}(0.73):<br>0 0 1 1 1 0 1 1 1 1 1 1 1 1 0 1 1 1 1 0 1 1 1 0 1 1 1 1 0 0<br>B_{LC}(0.5):<br>1 0 1 0 1 0 0 1 1 0 0 0 1 1 1 1 0 0 0 1 0 1 1 0 1 1 0 0 0 1	C_{HC}(0.73):<br>0 1 1 1 1 1 1 1 1 0 1 1 0 1 1 0 1 1 0 1 1 1 0 0 1 1 1 0 1 1<br>D_{HC}(0.2):<br>0 0 0 0 0 1 0 0 0 1 1 0 0 0 0 1 0 0 0 0 0 1 0 0 0 0 0 0 1 0
**p1.3**	A_{LC}(0.6):<br>1 0 1 0 1 0 1 0 1 1 1 1 0 0 1 0 1 1 1 0 1 1 0 1 1 0 0 1 0 1<br>B_{LC}(0.4):<br>0 1 0 0 0 1 0 0 1 1 0 1 1 0 0 1 0 1 0 0 1 0 0 1 0 1 0 1 0 0	C_{HC}(0.6):<br>0 1 1 0 0 1 1 1 0 1 0 1 0 1 1 0 1 1 1 0 1 0 1 0 1 1 0 1 0 1<br>D_{HC}(0.1):<br>0 0 0 0 1 0 0 0 0 0 0 0 0 0 1 0 0 0 0 0 0 0 1 0 0 0 0 0 0 0
**p2**	A_{LC}(0.7):<br>0 1 1 1 1 1 0 1 0 1 1 0 1 1 1 0 1 1 1 0 0 0 1 1 1 1 1 0 1 1<br>B_{LC}(0.5):<br>1 0 0 1 1 1 1 0 1 1 1 0 0 1 1 0 0 0 1 1 0 0 1 1 0 0 0 1 0 0	C_{HC}(0.7):<br>1 0 1 1 1 0 1 1 1 1 1 1 1 0 1 0 0 0 1 1 0 1 1 0 1 1 1 0 1 1<br>D_{HC}(0.2):<br>1 0 0 0 0 0 0 1 0 1 0 0 0 0 0 0 0 0 1 1 0 0 0 0 0 0 1 0 0 0
**p3**	A_{LC}(0.7):<br>1 1 0 1 0 1 1 1 0 1 0 1 1 1 0 1 1 1 1 0 0 0 1 0 1 1 1 1 1<br>1 1 0 1 1 0 1 0 1 1 1 1 1 0 0 1 0 1 1 1 1<br>B_{LC}(0.5):<br>1 0 0 1 0 1 1 1 1 0 1 1 1 0 0 1 1 0 0 0 0 0 0 1 0 1 1 1 0<br>0 1 1 0 1 1 1 0 0 0 1 0 1 0 1 1 0 0 1 0 0	C_{HC}(0.7):<br>0 1 1 0 1 1 1 0 1 1 1 0 1 0 1 1 1 1 0 1 0 1 1 0 1 0 1 1 1 1<br><br>D_{HC}(0.4):<br>1 0 0 0 1 0 1 0 0 1 0 1 0 1 1 0 0 1 0 0 0 1 0 1 1 0 0 1 0 0
**p4.1**	A_{LG}(0.6):<br>1 1 0 1 0 1 0 1 1 0 0 1 1 0 1 0 1 0 1 0 1 0 1 1 1 0 1 0 1 1<br>B_{LG}(0.4):<br>1 1 0 0 1 0 0 0 1 0 0 0 1 0 1 0 0 1 0 1 0 1 0 1 0 1 0 1 0 0	C_{HG}(0.76):<br>0 1 1 1 1 1 0 1 1 1 1 0 1 1 1 0 1 1 1 0 1 0 1 1 1 1 1 0 1 1<br>D_{HG}(0.56):<br>1 0 0 1 1 1 1 0 1 1 1 0 0 1 1 0 0 1 1 1 0 0 1 1 0 1 0 1 0 0
**p4.2**	A_{LG}(0.6):<br>0 1 0 1 0 1 0 1 1 0 0 1 1 0 1 0 1 1 1 0 1 0 1 1 1 0 1 0 1 1<br>B_{LG}(0.4):<br>1 1 0 0 1 0 0 0 1 0 0 0 1 0 1 1 0 1 0 0 1 1 0 0 1 0 0 1 0 0	C_{HG}(0.76):<br>1 1 1 1 1 1 0 1 1 0 1 0 1 1 1 0 0 0 1 1 1 1 1 1 1 1 1 0 1 1<br>D_{HG}(0.56):<br>0 0 0 1 1 1 1 0 1 1 1 0 0 1 1 0 1 0 1 1 1 1 0 1 0 0 1 1 0 0

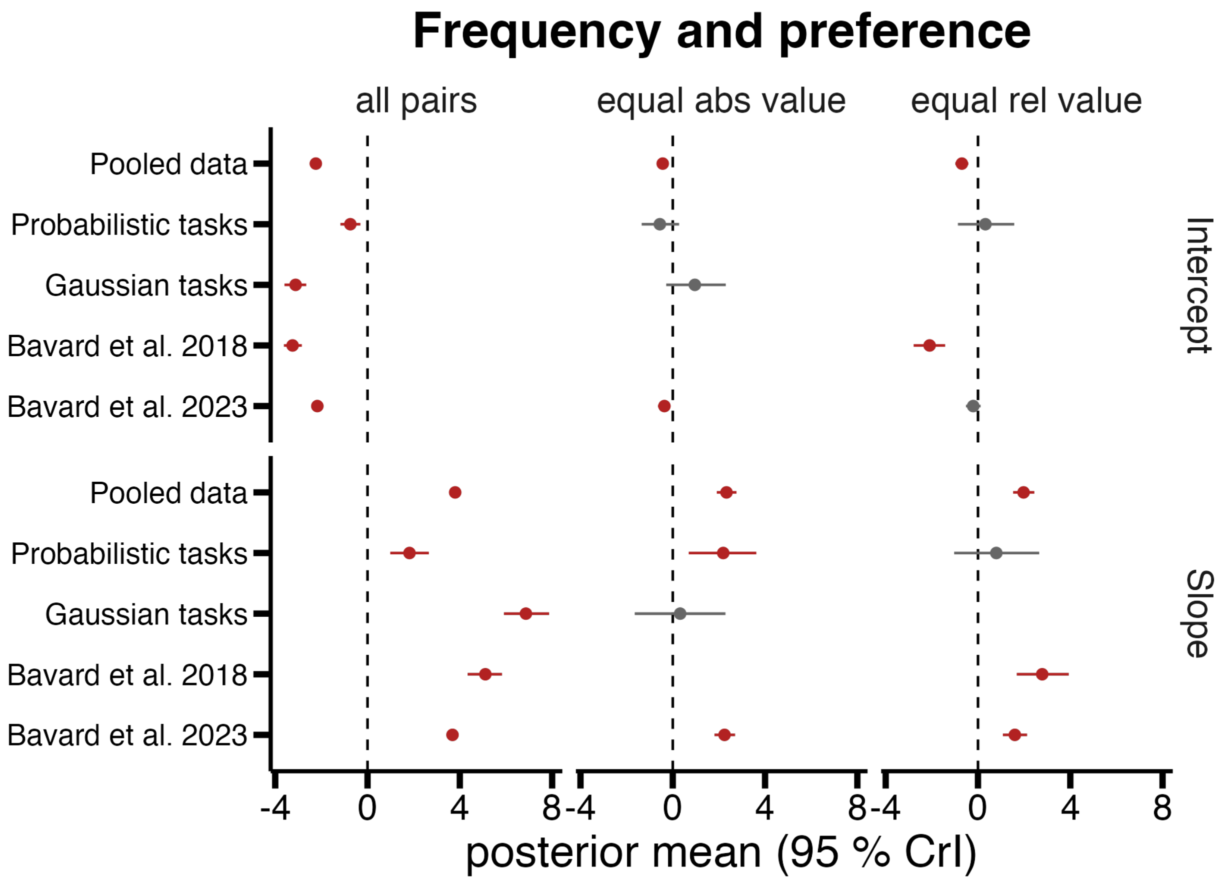

Supplementary Figure 1. Hierarchical regressions without partial feedback tasks. Facets in the columns separate the three data subsets we analyze ("all pairs", "equal absolute value", "equal relative value"), while rows distinguish Intercept versus Slope. Each horizontal line depicts the 95 % credible interval for the posterior mean of either the Intercept or the Slope. Points are posterior means; red intervals mark estimates whose 95 % CI excludes 0 (credible frequency effect), grey intervals those overlapping 0. The dashed vertical reference line at 0 aids visual inspection; intervals that lie entirely to the right indicate a positive repetition-to-preference relation. Note, to further control for experienced value both partial-feedback tasks (p2 and g2) are omitted, so all estimates are based exclusively on full-feedback datasets where all rewards were observable.

Participant level model diagnostics

For each hierarchical fit we extracted the stored point-wise log-likelihood matrix, sliced the appropriate columns for every participant's trials, and computed the participant level WAIC [8]. We then (1) summarized the participant distribution (first column in Supplementary Figure 2 and 3), (2) reported the percentage of participants best-fit by each model (second column in Fig. 2 and 3), and (3) repeated the classical group comparison with ΔWAIC (third column in Supplementary Figure 2 and 3). The repetition-bias (REP) model yields the lowest WAIC in most participants across most tasks and remains the global optimum at the group level except for tasks p4.2 and g2. While the advantage of the REP model is expressed at the individual level one can also see that in a subset of tasks the REL model also performs well at explaining participant behavior. However, we explicitly tested this in our task design and also show using simulations that the REL model digresses from its own predictions (see Supplementary Figure 6). Note that when computing the participant level WAIC from group level fits, these values are obtained after shrinkage, i.e. borrowing information through the group level parameters. However, we used uninformative priors on the group level parameters, and any possible shrinkage affects all models equally, so differences in WAIC per participant should remain meaningful[8].

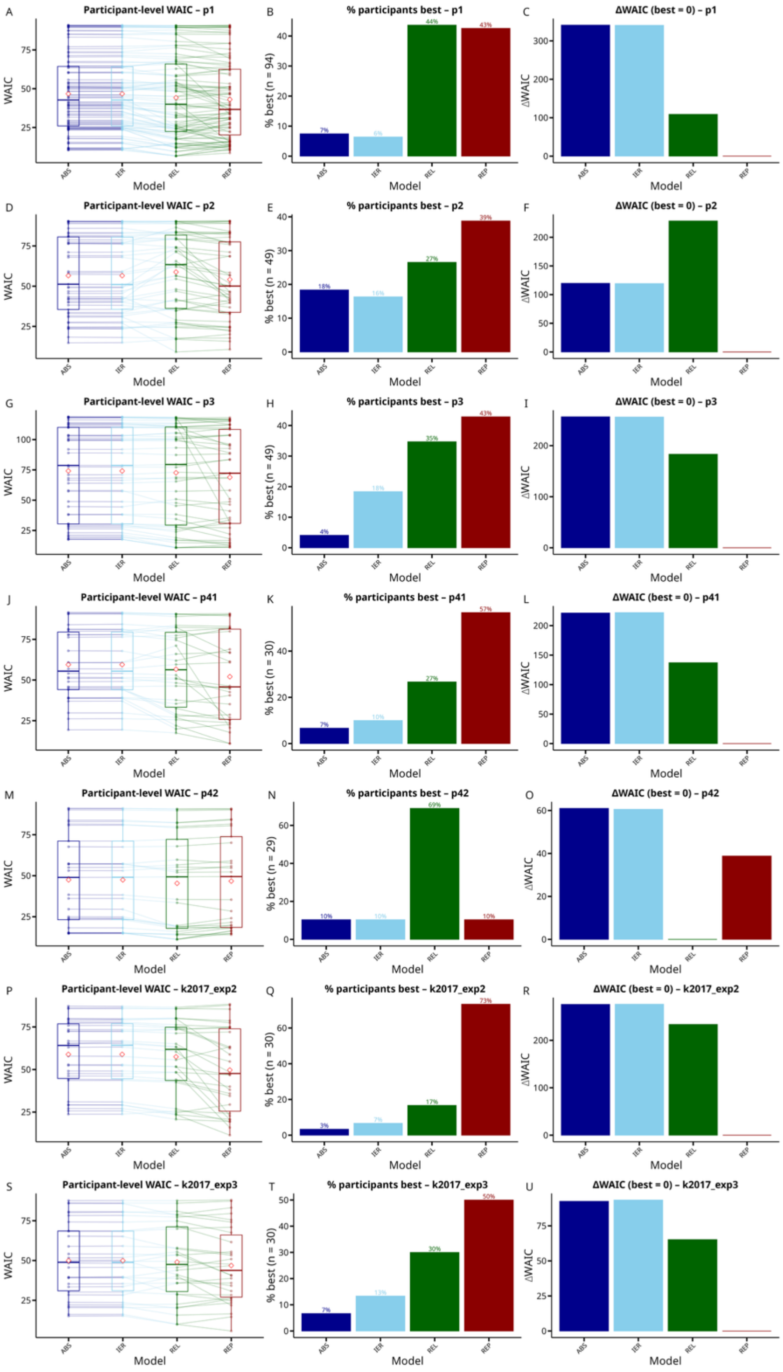

Supplementary Figure 2 Dataset-wise model comparison at the participant-level using WAIC. Each task is represented by a three-panel row (left to right). A, G, D, J, M, P, S: Individual WAIC: every thin line connects the WAIC values of one participant across the fitted models. Dots are the raw scores and the box-and-whisker plot summarises the distribution and the red diamond marks the sample mean. B, E, H, K, N, Q, T: Proportion best: bars show the percentage of participants for whom a given model achieved the lowest WAIC (numbers above the bars give the exact percentages). C, F, I, L, O, R, U: ΔWAIC: bar height equals the difference between the summed WAIC of a model and the overall best (lower is better; the optimum therefore appears at 0). WAIC was computed from the point-wise log-likelihood columns stored in the hierarchical MCMC output; for every participant we sliced the columns belonging to their trials and applied WAIC via the loo R package[8].

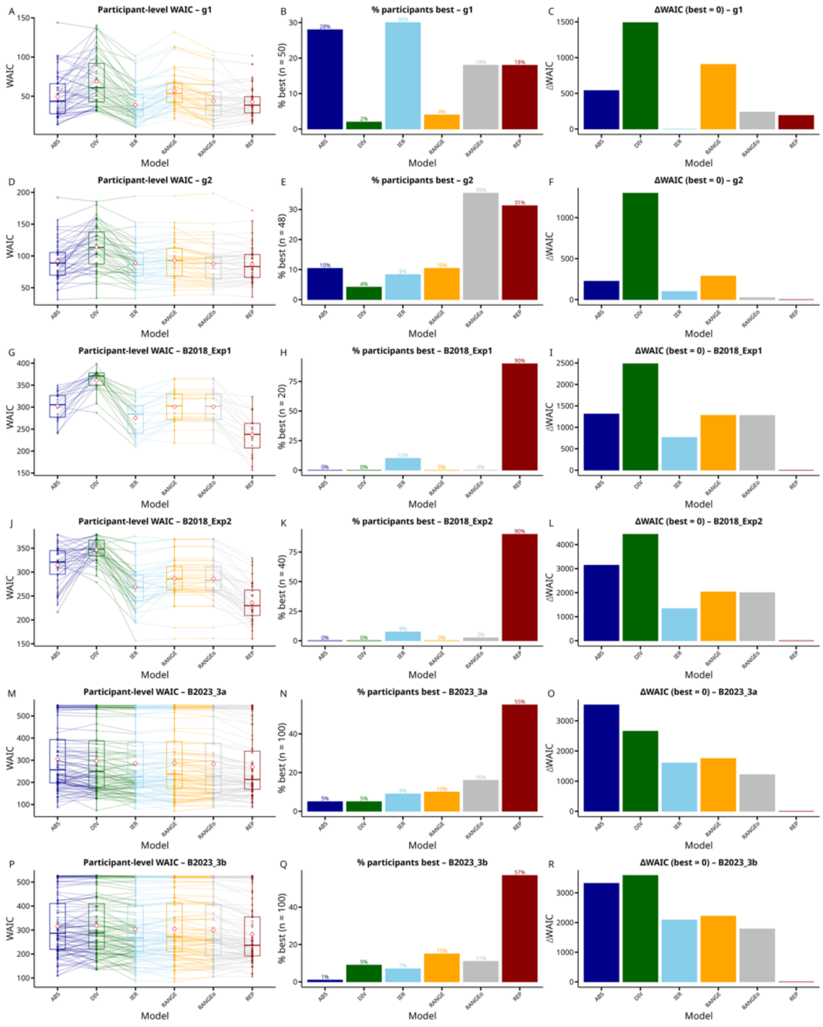

Supplementary Figure 3 Dataset-wise model comparison at the participant-level using WAIC. Each task is represented by a three-panel row (left to right). A, G, D, J, M, P: Individual WAIC, every thin line connects the WAIC values of one participant across the fitted models. Dots are the raw scores, the box-and-whisker plot summarises the distribution and the red diamond marks the sample mean. B, E, H, K, N, Q: Proportion best, bars show the percentage of participants for whom a given model achieved the lowest WAIC (numbers above the bars give the exact percentages). C, F, I, L, O, R: ΔWAIC, bar height equals the difference between the summed WAIC of a model and the overall best (lower is better; the optimum therefore appears at 0). WAIC was computed from the point-wise log-likelihood columns stored in the hierarchical MCMC output; for every participant we sliced the columns belonging to their trials and applied WAIC via the loo R package[8].

Parameter recovery

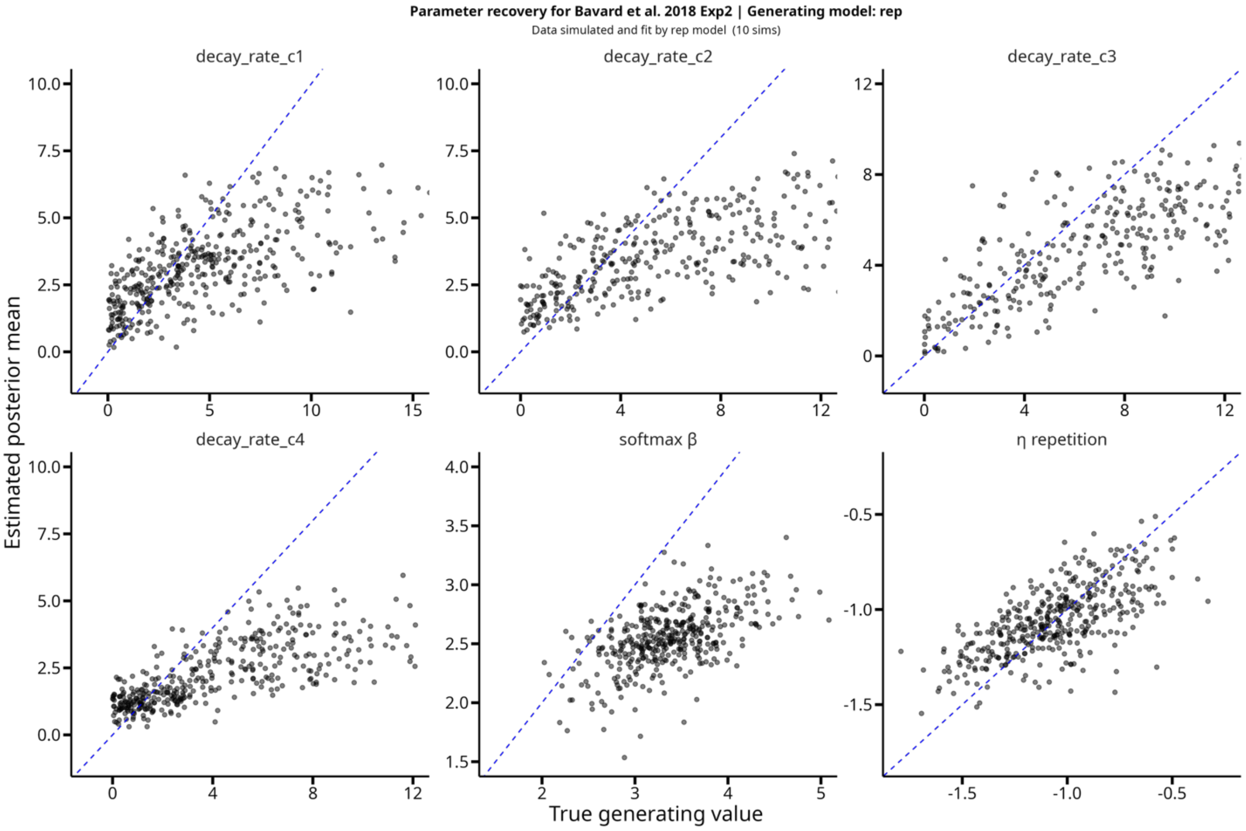

Supplementary Figure 4 Scatter plots of true generating values (x-axis) against posterior means after refitting (y-axis) for each parameter of the REP model. Each facet corresponds to one parameter. Points represent individual participants across 10 simulated full datasets. The dashed diagonal marks perfect recovery. Parameters showed moderate to good recovery, while notably parameter recovery worsens in the tail of the context specific learning decays. decay_rate_c1: r = 0.64, decay_rate_c2: r = 0.61, decay_rate_c3: r = 0.78, decay_rate_c4: r = 0.59 softmax β: r = 0.57, η repetition: r = 0.67. Note that because simulating directly from the posterior we account for the whole within and between subject variance-covariance structure.

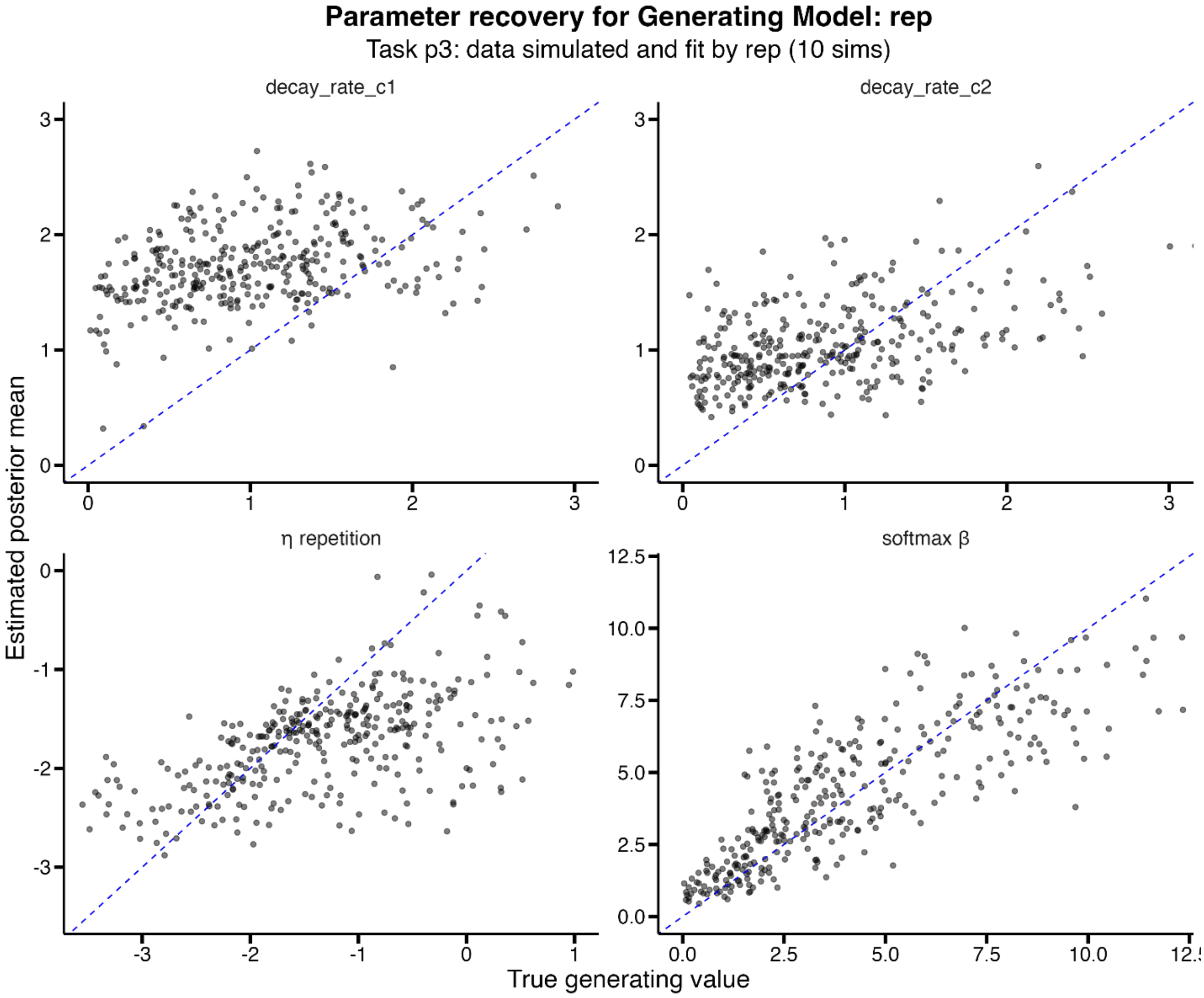

Supplementary Figure 5 Scatter plots of true generating values (x-axis) against posterior means after refitting (y-axis) for each recoverable parameter of the REP model. Each facet corresponds to one parameter. Points represent individual participants across 10 simulated full datasets. The dashed diagonal marks perfect recovery. Parameters showed moderate to good recovery, while notably parameter recovery worsens in the tail of the context specific learning decays. decay_rate_c1: r = 0.31; decay_rate_c2: r =0.53; softmax β: r =0.80, η repetition: r =0.58. Note that because simulating directly from the posterior we account for the whole within and between subject variance-covariance structure.

Model recovery

We performed full model confusion for a range of representative tasks that differ in their reward generating process (probabilistic or Gaussian), model predictions and task complexity. In our procedure, we generated posterior-predictive data during MCMC: at each post–warm-up draw we simulated a full dataset from the likelihood conditional on that joint parameter draw. Because our sampler stored 10,000 post-warm-up draws, this yielded 10,000 posterior-predictive datasets for each model. For the recovery analysis, we then randomly selected 20 (per simulated model) of these datasets and re-fit each once with every candidate model using the same MCMC settings as in the main analysis. This approach preserves within-subject and cross-parameter covariance learned from the empirical data and yields recovery results that are most relevant to the empirically plausible parameter region (while accounting for within-subject parameter correlations and uncertainty).The NxN confusion matrix shows how often each model on the group level best fitted the data of each generating model.

Note that our model-recovery analysis is conducted at the group level within a hierarchical Bayesian framework. Aggregating across simulated participants can increase separation between models and may overestimate subject-level discriminability. Individual fits can show heterogeneity (see Supplementary Figure 2 and 3), and some participants may be better captured by alternative models. Accordingly, the recovery results should be interpreted as evidence for group-level discriminability rather than a claim that a single model uniformly explains every individual.

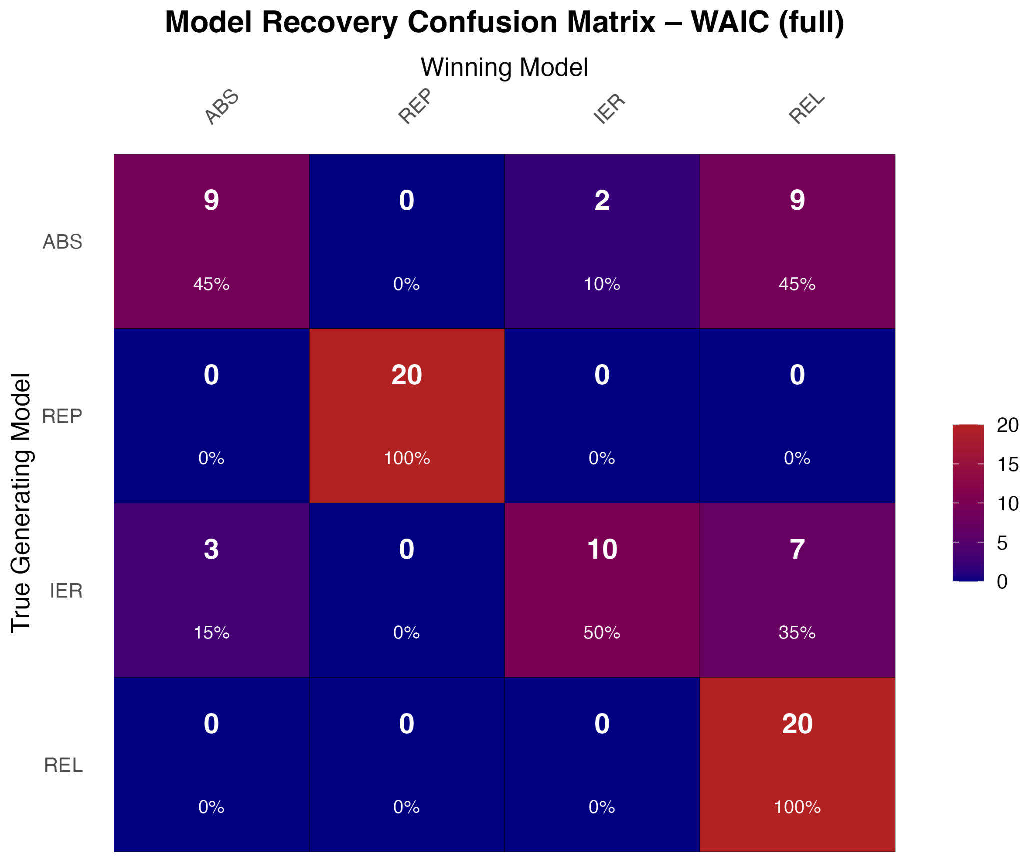

Supplementary Figure 6 Confusion matrix for task p3. Results show a full model recovery analysis using WAIC scores for 20 simulated full datasets (all participants and trials) per model. Each cell indicates how often a dataset generated by a given model (rows: "True Generating Model") was best fit by one of the candidate models (columns: "Winning Model"). Both REP and REL models are correctly identified in 100% of cases, suggesting they are distinguishable from competing models given the task structure and number of trials. In contrast, the ABS and IER models show confusion. Data generated by ABS is often misattributed to REL (45%) or IER (10%), while IER simulations are frequently misclassified as REL (35%) or ABS (15%). This pattern suggests that, in this probabilistic task, REP and REL models are identifiable, while ABS and IER may produce qualitatively similar behavior. These results are consistent with our theoretical expectations, as ABS and IER make similar predictions in this probabilistic task, and REL (with decaying learning rates) often fits the data well despite violating its own theoretical predictions (see Simulations below).

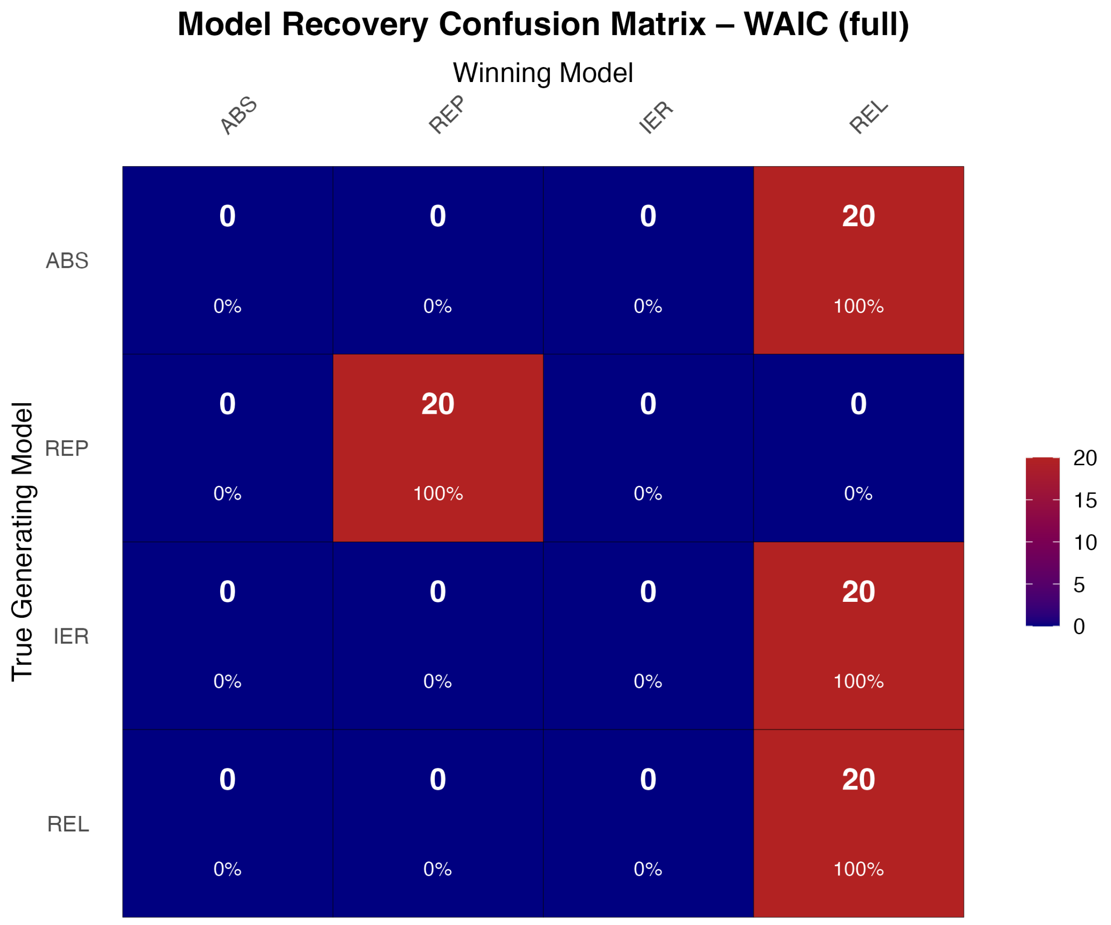

Supplementary Figure 7 Confusion matrix for task p4.1. Results show a full model recovery analysis using WAIC scores for 20 simulated full datasets (all participants and trials) per model. REP and REL models are again identifiable, each correctly recovered in 100% of simulations. In contrast, both ABS and IER models are consistently misclassified as REL. This likely reflects two factors: first, ABS and IER make highly similar predictions under those task dynamics; second, the REL model is comparatively simpler and can accommodate the behavior generated by ABS and IER with fewer parameters (see simulations below). These results suggest that while REP and REL produce clearly distinguishable behavioral patterns in task p4.1, the ABS and IER models lack uniquely identifiable signatures, either with each other or with the more parsimonious REL model.

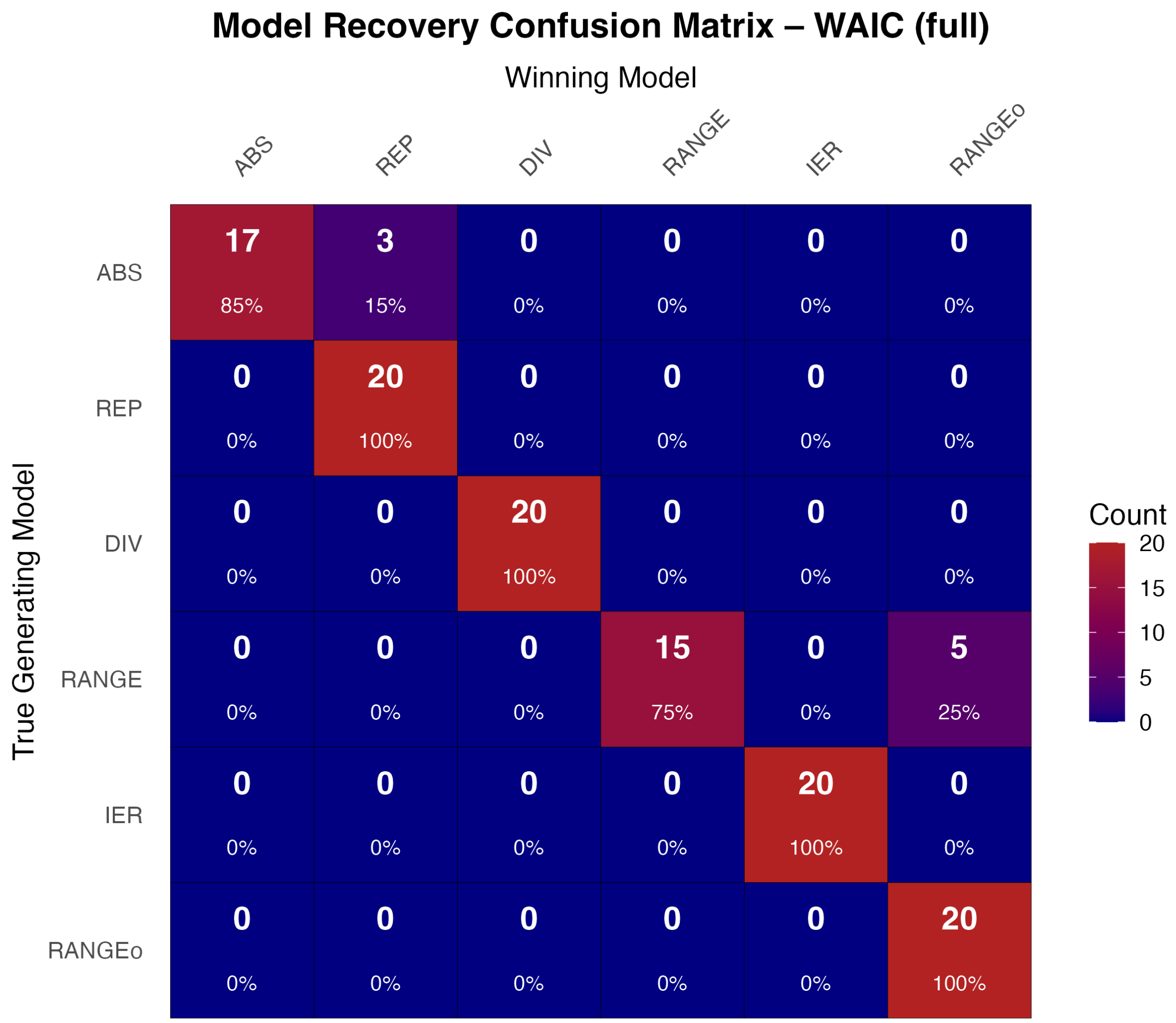

Supplementary Figure 8 Confusion matrix for task g1. Results show a full model recovery analysis using WAIC scores for 20 simulated full datasets (all participants and trials) per model for ABS, REP, DIV, RANGE, IER, and RANGEo models. All models except RANGE and ABS show high identifiability, with 100% correct recovery. RANGE was recovered in 75% of cases, with the remaining 25% misclassified as RANGEo, a more complex variant. This partial confusability highlights the similarity of predictions between RANGE and RANGEo. Further 15% of ABS datasets were better fit by the REP model, which is likewise a more complex version of the ABS model. Results show that most models are identifiable in this task, though some value normalization variants (RANGE vs. RANGEo) remain more difficult to distinguish given this task.

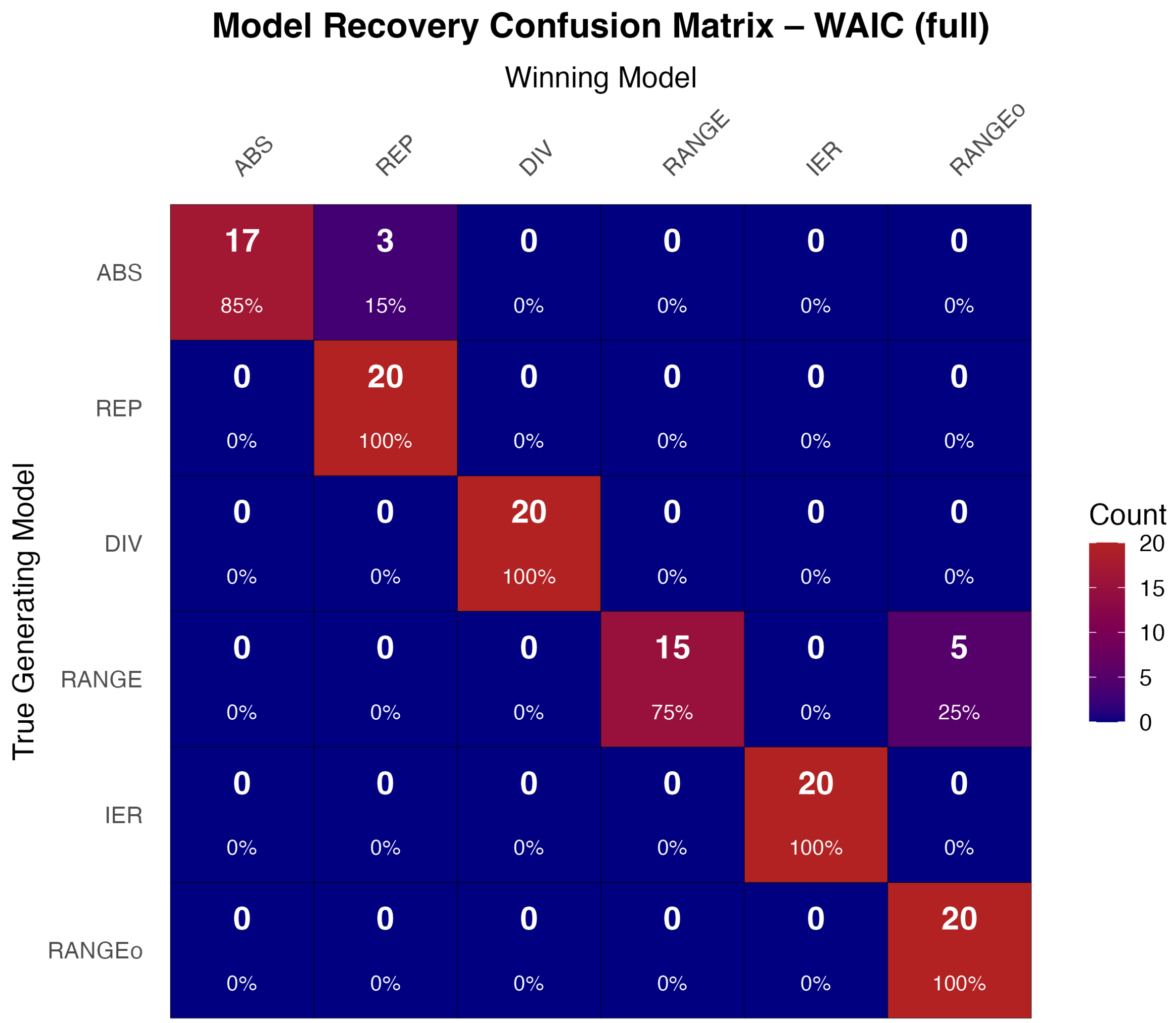

Supplementary Figure 9 Confusion matrix for Exp 2 from Bavard et al. 2018[9]. Results show a full model recovery analysis using WAIC scores for 20 simulated full datasets (i.e., all participants and trials) per model for ABS, REP, DIV, RANGE, IER, and RANGEo models. All models except RANGE and ABS show high identifiability, with 100% correct recovery. RANGE was recovered in 75% of cases, with the remaining 25% misclassified as RANGEo, a more complex variant. This partial confusability highlights the similarity of predictions between RANGE and RANGEo. Further 15% of ABS datasets were better fit by the REP model, which is likewise a more complex version of the ABS model. Results show that most models are identifiable in this task, though some value normalization variants (RANGE vs. RANGEo) remain more difficult to distinguish given this task.

Model simulations

To assess the qualitative predictions of our models, we conducted simulation-based analyses focusing on three newly collected datasets (p3, p4.1, and p4.2), in which predictions from the REL and REP models diverge most strongly. These simulations provide insight into whether a given model can simultaneously reproduce observed learning-phase dynamics and transfer preferences. Here Supplementary Figure 10 shows the evolution of latent value trajectories (Q-values, C-values [combined Q- and choice values], and Q-value differences), and Supplementary Figure 11 shows group-level transfer choice proportions across model simulations and empirical data. For each model (ABS, IER, REL, REP), we simulated 15 complete datasets, each spanning 50 participants. We set group-level means to decay $\delta = 0.8$, learning rate $\alpha = 1.0$, softmax inverse-temperature $\beta = 5$, and repetition weight $\eta = 0.4$. Subject-level parameters were then drawn around these means (using modest standard deviations) to induce realistic between-participant variability. These forward simulations generated latent trial-by-trial estimates: plain Q-values for ABS, combined C-values for REP (i.e., Q + choice values), and Q-differences for REL. To benchmark these predictions against human behavior, we also plotted latent trajectories from the same models when fitted to actual participant data, showing posterior-predictive dynamics under posterior parameters.

Across all three tasks, the REP model closely matches the observed learning-phase dynamics and replicates the predicted transfer phase preferences (see Supplementary Figure 10 and 11). The REL model, by contrast, systematically predicts preference for the opposite stimulus or indifference unless it post-hoc (in fitted trajectories only) adjusts context-specific gaps that violate its own assumptions. While REL and REP can fit the same data in quantitative terms, the REP model does so while also preserving theoretical consistency and producing qualitatively valid predictions under forward simulation. Notably, the ABS and IER models fail to capture both learning dynamics and transfer patterns, especially in cross-context comparisons. These models predict symmetrical or undifferentiated value trajectories that do not map onto observed behavior. We emphasize that simulation-based evaluations should be interpreted cautiously. While our models have similar complexity, their predictions depend on specific assumptions, e.g., symmetric vs. asymmetric learning rates, separate or shared decay across contexts and others. If a model can adjust its assumptions during fitting, it might also do so during simulation, if simulated with a specific combination of parameters. This general issue is however not unique to our work and affects prior simulation-based validations and studies in the field.

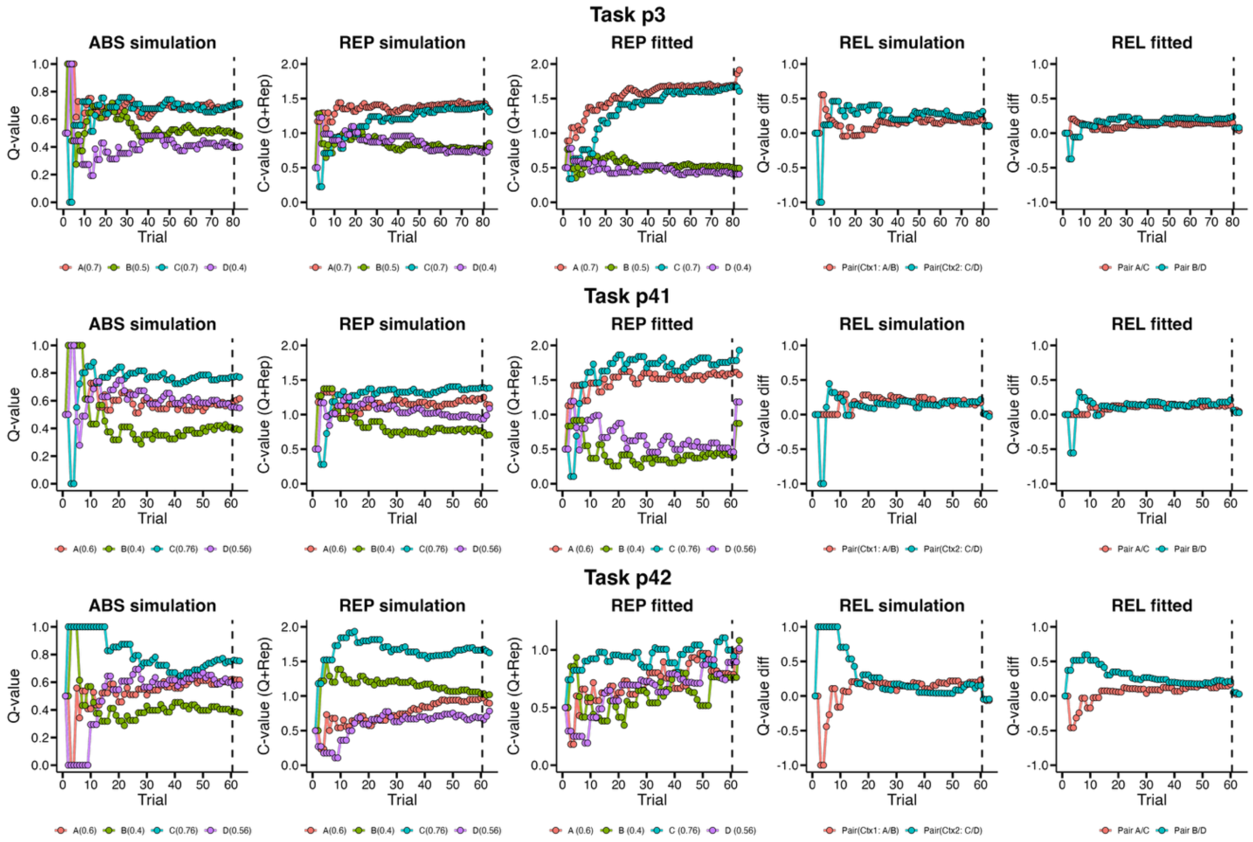

Supplementary Figure 10 Each row depicts one task in which the candidate models diverge qualitatively: p3 (top), p4.1 (middle) and p4.2 (bottom). Columns show, from left to right: ABS simulation, REP simulation, REP fitted to experimental data, REL simulation, and REL fitted to experimental data. Dots are trial-wise group means. Here only the REP model simultaneously reproduces both learning-phase dynamics that are in line with the observed transfer choices in all three tasks (see. Figure 2 in the main manuscript). In task p3 (row one) the REP simulation correctly predicts higher average combined values for stimulus A. That is this stimulus, despite its lower relative value in the LC context, on average overtakes stimulus C during learning, which we also see in the averaged fitted Q- and choice values (see top row, column two and three). In contrast, the REL simulation learns higher relative values for the best stimulus C in context 2 and would therefore predict preference for this stimulus (see column four in the first row). In task p4.1 the REP simulation (and fit) assigns less divergent combined values to stimulus A (red) and C (blue) than in task p4.2, where the REP simulation captures the larger between-context gap that drives a strong preference for the best HG stimulus. The REL model predicts indifference for simulations in both tasks and can only mimic a preference C (blue) by learning a bigger value difference in context 2 (HG) than in context 1 (LG; see column five in the bottom row). This is an ad-hoc departure from its own theoretical logic, which would predict indifference otherwise. Thus, across these tasks, the repetition-bias mechanism is the only mechanism whose neutral forward predictions align with the fitted latent values and with participants' transfer preferences (see Figure 2 in the main manuscript and Supplementary Figure 11 below).

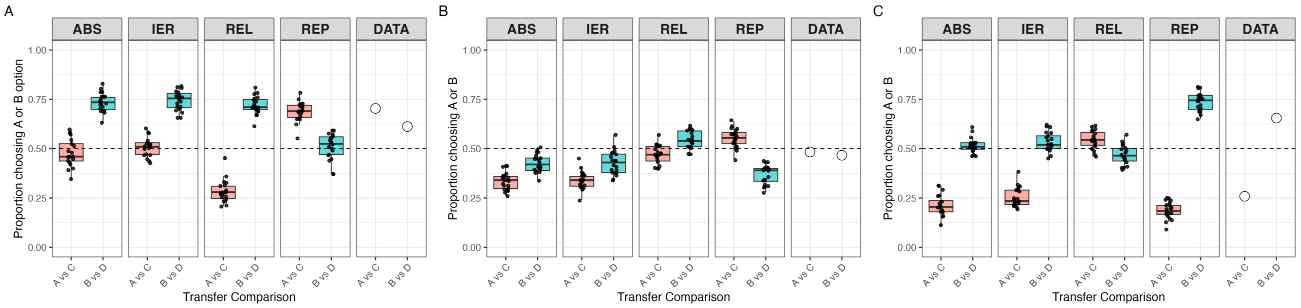

Supplementary Figure 11 Transfer phase predictions. Panels A–C show simulated choice proportions from each model (ABS, IER, REL, REP) alongside the observed data (DATA) for A: task p3, B: task p4.1, and C: task p4.2. Each dot represents the proportion of choices favoring one option over another (A vs C in red; B vs D in blue), computed across the full group of participants for one of 15 full-dataset simulations per model. The dashed line at 0.5 indicates no difference. Only the REP model consistently captures the direction and magnitude of observed transfer preferences across those tasks.

Different learning mechanisms

We compared two distinct learning mechanisms in our study. First, following Sutton and Barto[10], we implemented decaying learning rates, which are appropriate when reward probabilities (bandits) are stationary. Second, consistent with prior research[2,6,9], we fitted separate constant learning rates for chosen and unchosen options. Overall, models with context-specific decaying learning rates outperformed models with different learning rates for chosen and unchosen options in 30 out of 40 models applied to probabilistic task data and in 9 out of 24 models applied to Gaussian task data.

Repetition bias

In the REP model, the repetition learning rate parameter $\eta_{\mathrm{repetition}}$ primarily ranged from 0.03 to 0.25 across 12 of the 15 analyzed tasks (see Supplementary Table 7 for summary statistics and Supplementary Figure 1 for visualization). Although this parameter varied with different task parameterizations, it remained relatively consistent across different participant groups performing identical tasks under the same conditions (see Supplementary Figures 1B, 1D, and 1E). We interpret this stability as evidence of a common underlying mechanism driving choice behavior. While the height of the parameter is likely a function of task dynamics as the repetition parameter does not only model transfer preferences but also behavior during the learning phase.

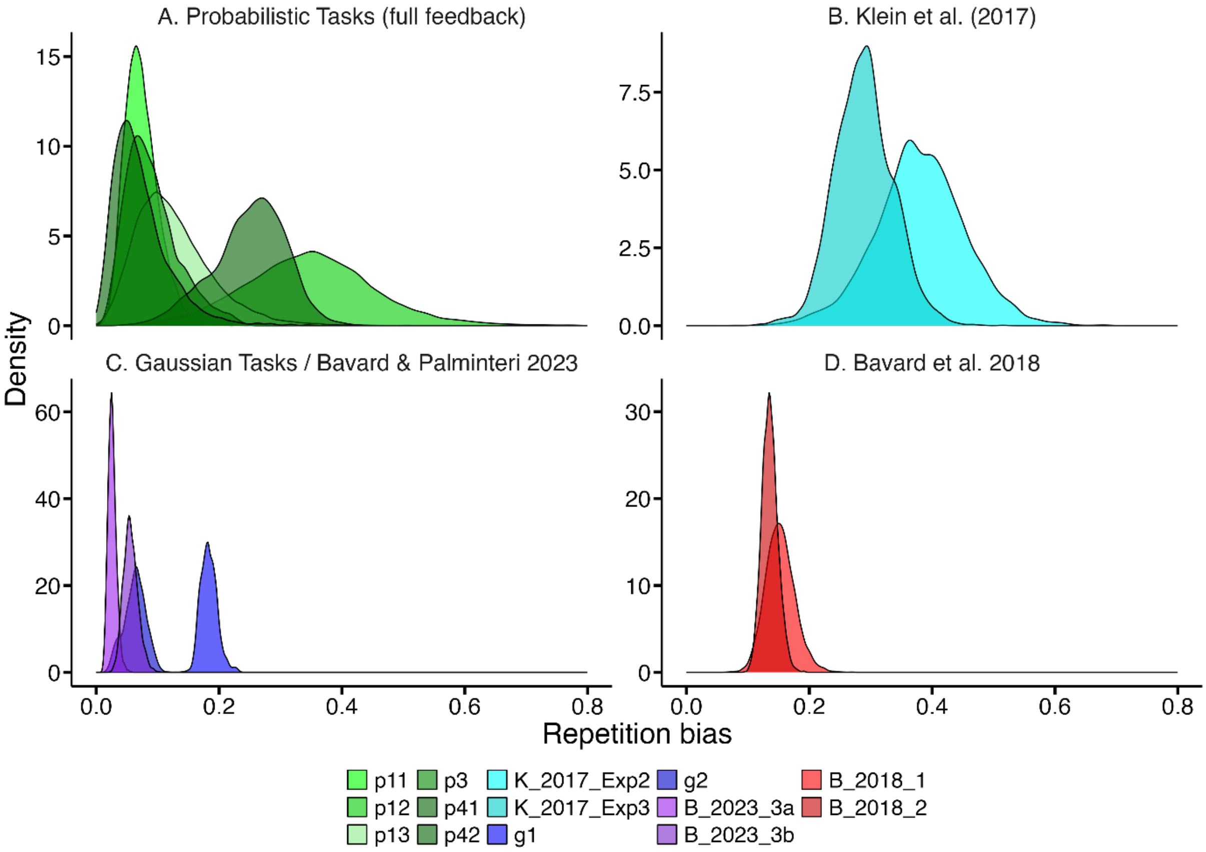

Supplementary Figure 12 $\eta_{repetition}$ varies between different datasets but remains relatively stable among participant groups performing identical tasks. For example, most parameter estimates for probabilistic tasks range from 0.05 to 0.2. Tasks from Klein et al. 2017[2] exhibit substantial overlap in their $\eta_{repetition}$ hyperparameter distributions, while datasets from Bavard and Palminteri[6] and Bavard et al.[9] display similar repetition-bias parameters (also see Supplementary Table 5).

Supplementary Table 5 95% highest density interval (HDI) and mean values for $\eta_{repetition}$ hyperparameter distributions across all analyzed datasets.

**Task**	**p1**	**p2**	**p3**	**p4.1**	**p4.2**	**Klein et al. 2017 Exp2**	**Klein et al. 2017 Exp3**
**95% HDI (lower-upper)**	0.0959-0.2535	0.0013-0.0196	0.0207-0.1809	0.1270-0.3579	0.0029-0.1595	0.2459-0.5389	0.1997-0.3834
**Mean**	0.1722	0.0064	0.0915	0.2498	0.0726	0.3810	0.2891
**Task**	**g1**	**g2**	**Bavard et al. 2018 Exp1**	**Bavard et al. 2018 Exp2**	**Bavard and Palminteri 2023 Exp3a**	**Bavard and Palminteri 2023 Exp 3b**	
**95% HDI (lower-upper)**	0.1582-0.2098	0.0264-0.0947	0.1067-0.2003	0.1105-0.1614	0.0143-0.0391	0.0347-0.0804	
**Mean**	0.1835	0.0630	0.1525	0.13597	0.02601	0.0561	

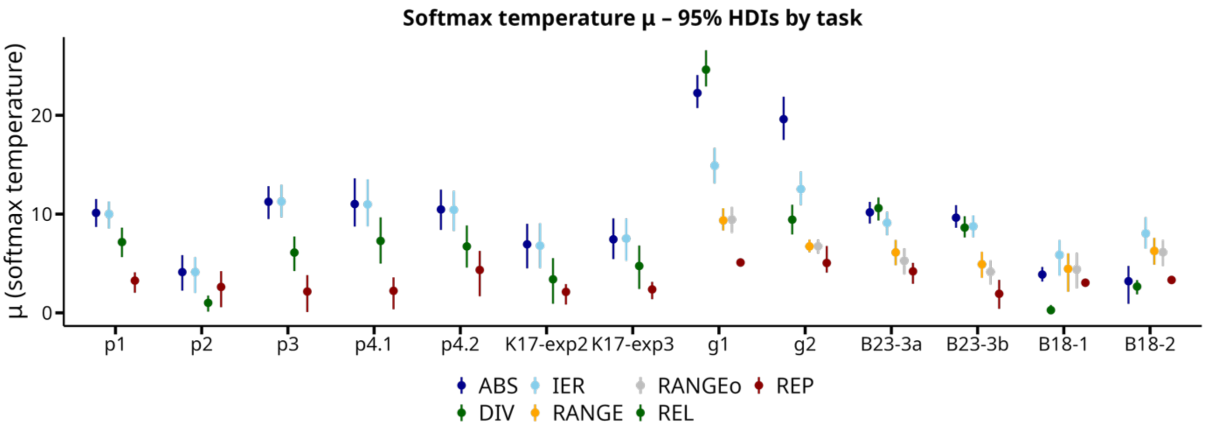

Supplementary Figure 13 Softmax β hyperparameter means across tasks and models. Each point represents the posterior median of the group-level softmax β parameter, with vertical lines indicating 95% highest density intervals (HDIs). Tasks are shown on the x-axis, color-coded by model. Higher values indicate steeper choice functions and increased sensitivity to value differences.

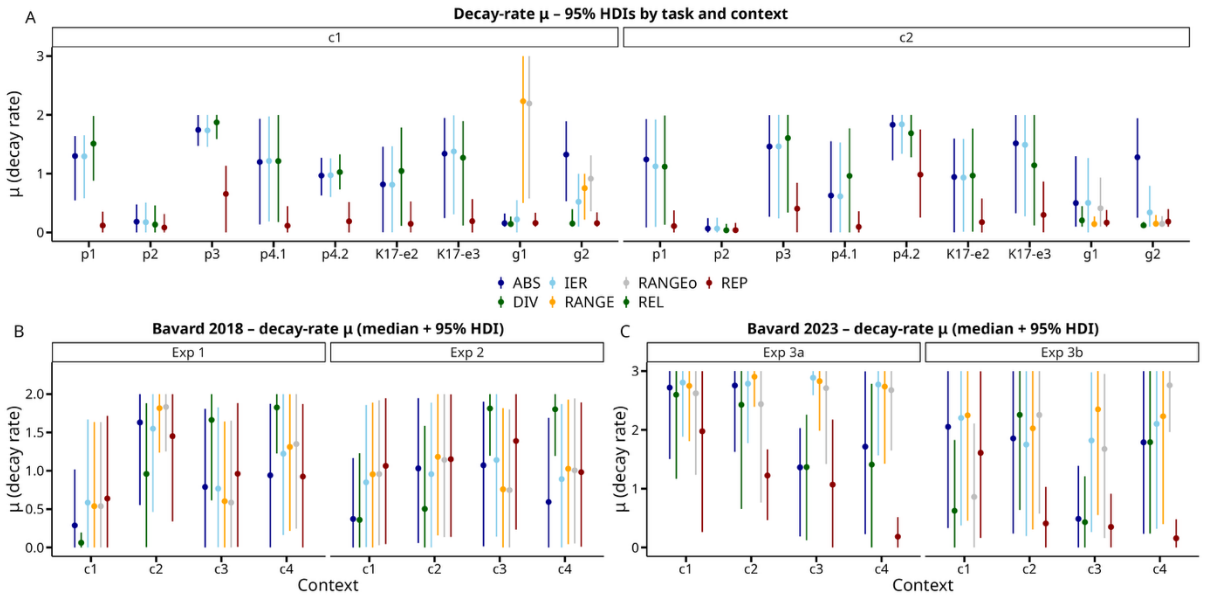

Supplementary Figure 14 Decay-rate hyperparameter means for each context across tasks and models. Points show posterior medians and 95% HDIs of the decay parameter for each learning context (c1–c4), reflecting how quickly the learning rate decays as a function of how often a context has been visited. Facets separate context (upper row) or experimental tasks (bottom row); colors denote the computational model. Differences across contexts indicate context-sensitive value learning.

Supplementary Table 6 Overview of group level WAIC fits and SE for all hierarchical models with context specific decaying learning rates

Task	Model	WAIC	SE_WAIC
B2018_Exp1	ABS	6,053.4	65.8
B2018_Exp1	IER	5,506.4	75.4
B2018_Exp1	DIV	7,225.5	56.0
B2018_Exp1	RANGE	6,022.3	67.8
B2018_Exp1	RANGEo	6,019.7	67.9
B2018_Exp1	REP	**4,742.0**	**82.4**
B2018_Exp2	ABS	12,581.6	86.6
B2018_Exp2	IER	10,777.5	106.0
B2018_Exp2	DIV	13,867.6	62.1
B2018_Exp2	RANGE	11,469.2	104.9
B2018_Exp2	RANGEo	11,440.2	105.2
B2018_Exp2	REP	**9,438.1**	**115.3**
B2023_3a	ABS	30,560.2	227.5
B2023_3a	IER	28,633.4	217.7
B2023_3a	DIV	29,688.0	226.2
B2023_3a	RANGE	28,782.8	221.7
B2023_3a	RANGEo	28,244.7	222.0
B2023_3a	REP	**27,030.1**	**206.4**
B2023_3b	ABS	31,585.3	223.3
B2023_3b	IER	30,353.5	217.1
B2023_3b	DIV	31,852.1	224.7
B2023_3b	RANGE	30,480.6	220.1
B2023_3b	RANGEo	30,049.1	222.9
B2023_3b	REP	**28,262.9**	**208.5**
g1	ABS	2,487.6	108.7
g1	IER	**1,948.3**	**91.8**
g1	DIV	3,438.6	117.9
g1	RANGE	2,854.7	100.2
g1	RANGEo	2,185.9	101.9
g1	REP	2,139.6	92.4
g2	ABS	4,400.8	109.2

Task	Model	WAIC	SE_WAIC
g2	IER	4,274.9	109.4
g2	DIV	5,474.8	127.8
g2	RANGE	4,463.2	112.1
g2	RANGEo	4,199.0	111.0
g2	REP	**4,175.5**	**110.3**
k2017_exp2	ABS	1,766.8	44.2
k2017_exp2	IER	1,767.2	44.2
k2017_exp2	REL	1,724.6	46.3
k2017_exp2	REP	**1,491.0**	**47.9**
k2017_exp3	ABS	1,497.7	49.7
k2017_exp3	IER	1,498.7	49.8
k2017_exp3	REL	1,470.4	53.7
k2017_exp3	REP	**1,405.2**	**49.4**
p1	ABS	4,389.7	80.5
p1	IER	4,389.1	80.4
p1	REL	4,157.6	79.9
p1	REP	**4,048.8**	**81.1**
p2	ABS	2,771.5	57.9
p2	IER	2,771.0	57.9
p2	REL	2,880.2	56.8
p2	REP	**2,651.8**	**58.2**
p3	ABS	3,631.8	60.0
p3	IER	3,631.4	60.0
p3	REL	3,558.4	63.5
p3	REP	**3,375.2**	**65.5**
p41	ABS	1,783.4	42.4
p41	IER	1,784.1	42.3
p41	REL	1,699.1	43.6
p41	REP	**1,562.0**	**46.2**
p42	ABS	1,377.2	40.3
p42	IER	1,376.8	40.3
p42	REL	**1,316.3**	**41.0**
p42	REP	1,355.1	42.6

Control analyses for relative frequency during learning and transfer preference

To test whether learned values (at the end of the learning phase), in the form of absolute or relative Q-values, that is their differences can account for transfer preference or explain away the frequency effect, we conducted the following control analyses: We first refitted our hierarchical ABS and REL RL models for each newly collected dataset (tasks p1, p2, p3, p4.1, p4.2, g1, g2) and the two datasets from Klein et al.[2]. Specifically, we used a model with different learning rates for chosen and unchosen options (ABS model) or different learning rates for each context (REL model). We then directly simulated Q-values from the joint posterior distribution at the end of the learning phase and (i) show these in side-by-side plots for both options with equal absolute values, i.e., option C in the high-contrast (HC) context and option A in the low-contrast (LC) context (tasks p1, p2, p3, g1, g2, and Exp 2 and Exp 3 from Klein et al., 2017) and for both options with equal relative values - option C in the high gain (HG) context and option A in the low gain (LG) context. Plotting these values shows that our task design (balanced and controlled reward schedule) worked as intended. Q-values at the end of learning show only minor variability (Supplementary Figures 15A, 16A, and 17A). Next, we show associations between Q-value difference and relative choice frequency (Supplementary Figures 15B,C; 16B,C; and 17B,C). If the association between relative choice frequency and transfer preference stemmed from differences in learned values, there should be a positive association between Q-value differences (option C [D] minus option A) and relative choice frequency (option C [D] versus option A). However, such an association is mostly absent. The only task showing a positive association is task g2 ($t(96) = 3.5987$, $p = 0.001$), driven by an extreme outlier (> 3.5 SD); if this data point is removed ($t(95) = 1.348$, $p = 0.18$), all correlations are non-significant. Likewise, recomputing both hierarchical regressions for equal absolute and equal relative comparisons does not change the results. Please note that these control analyses are not exhaustive and are not intended to be. We do not examine all tested associations (comparisons, datasets, e.g., both datasets from Bavard et al.[9], and Bavard and Palminteri[6]) and we do not use other forms of normalized values to control for effects on transfer preference. Therefore, this analysis should only be interpreted for what it is: evidence of an association with choice frequency, not an exclusive mechanistic explanation.

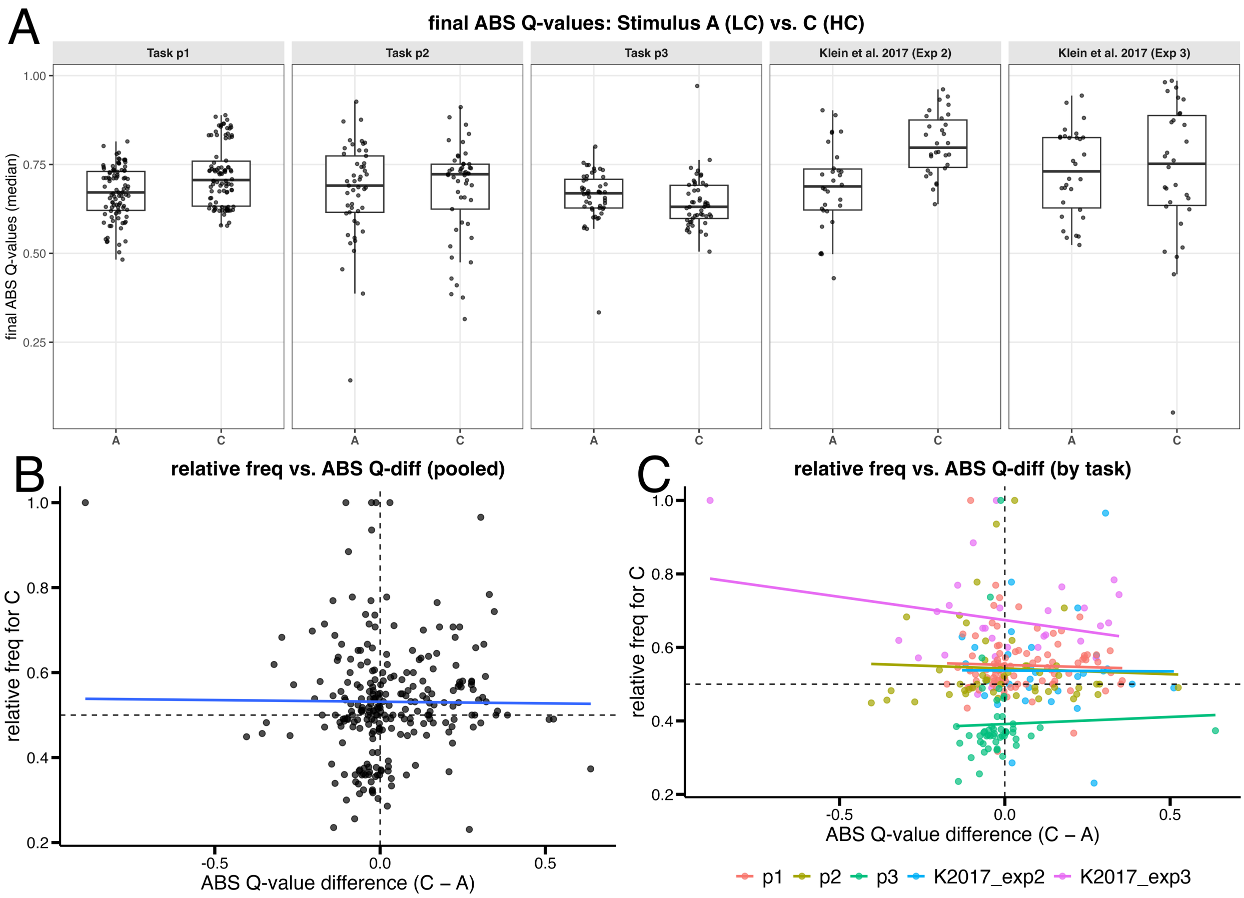

Supplementary Figure 15 **A:** Posterior-median absolute Q-values for all equal absolute value pairs across our new probabilistic tasks. Option A (0.7. LC) vs. option C (0.7 HC), shown separately for each task (Task p1, Task p2, Task p3, Klein et al. 2017 (Exp 2), Klein et al. 2017 (Exp 3)). Points are absolute Q-values at the end of the learning phase for individual participants estimated from the ABS RL model with different learning rates for chosen and unchosen options. **B:** Relationship between the ABS Q-value difference (x-axis; option C – option A) and the relative choice frequency of choosing C (in contrast to A) during learning (y-axis). The solid line is a linear fit; dashed lines mark the reference values where the difference in Q-values is 0 and relative frequency is 0.5 **C:** Same relationship as in panel B but split by task (points colored by task) with separate linear fits per task. We do not find any positive association between relative choice frequency and Q-value difference.

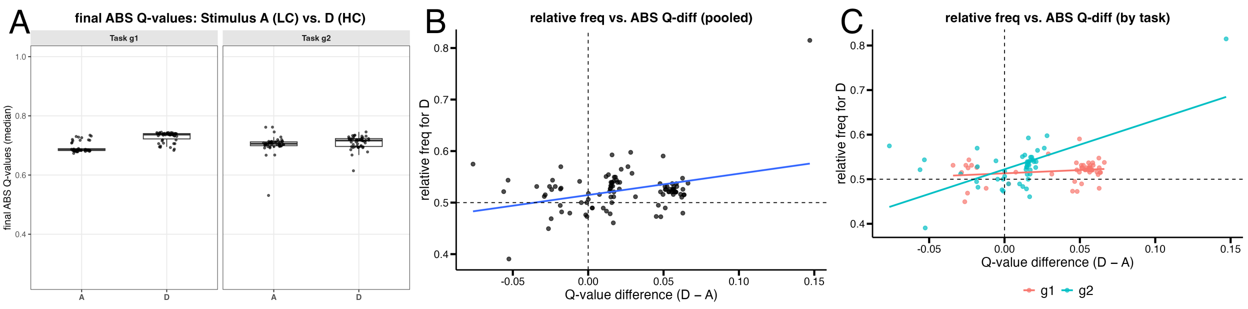

Supplementary Figure 16 **A:** Posterior-median absolute Q-values for all equal absolute value pairs across our new Gaussian tasks. Option A (LC) vs option D (HC), shown separately for Task g1 and Task g2. Points are absolute Q-values at the end of the learning phase for individual participants estimated from the ABS RL model with different learning rates for chosen and unchosen options. **B:** Relationship between the ABS Q-value difference (x-axis; option D – option A) and the relative choice frequency of choosing D (in contrast to A) during learning (y-axis). The solid line is a linear fit; dashed lines mark the reference values where the difference in Q-values is 0 and relative frequency is 0.5 **C:** Same relationship as in panel B but split by task (points colored by task) with separate linear fits per task. There is a positive association of relative choice frequency and Q-value difference in task g2. However, this is driven by one extreme outlier (> 4 SD; upper right) and n.s. when this datapoint is excluded.

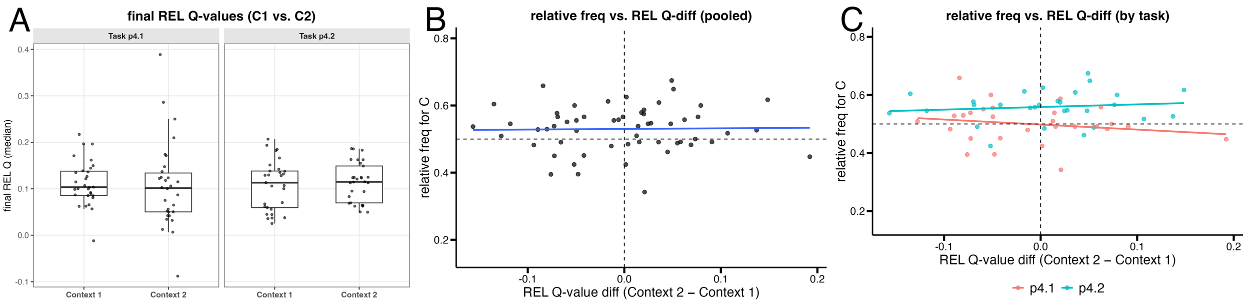

Supplementary Figure 17 **A:** Posterior-median relative Q-values for Context 1 and Context 2, shown separately for Task p4.1 and Task p4.2. Points are relative Q-values at the end of the learning phase for individual participants estimated from the REL RL model with different learning rates for each context. **B:** Relationship between the REL Q-value difference (x-axis; Context 2 – Context 1) and the relative choice frequency of choosing C (in contrast to A) during learning (y-axis). The solid line is a linear fit and dashed lines mark the reference values where the difference in relative Q-values is 0 and relative frequency is 0.5 **C:** Same relationship as in panel **B** but split by task (points colored by task) with separate linear fits per task. There is no positive association of relative choice frequency and relative Q-value difference.

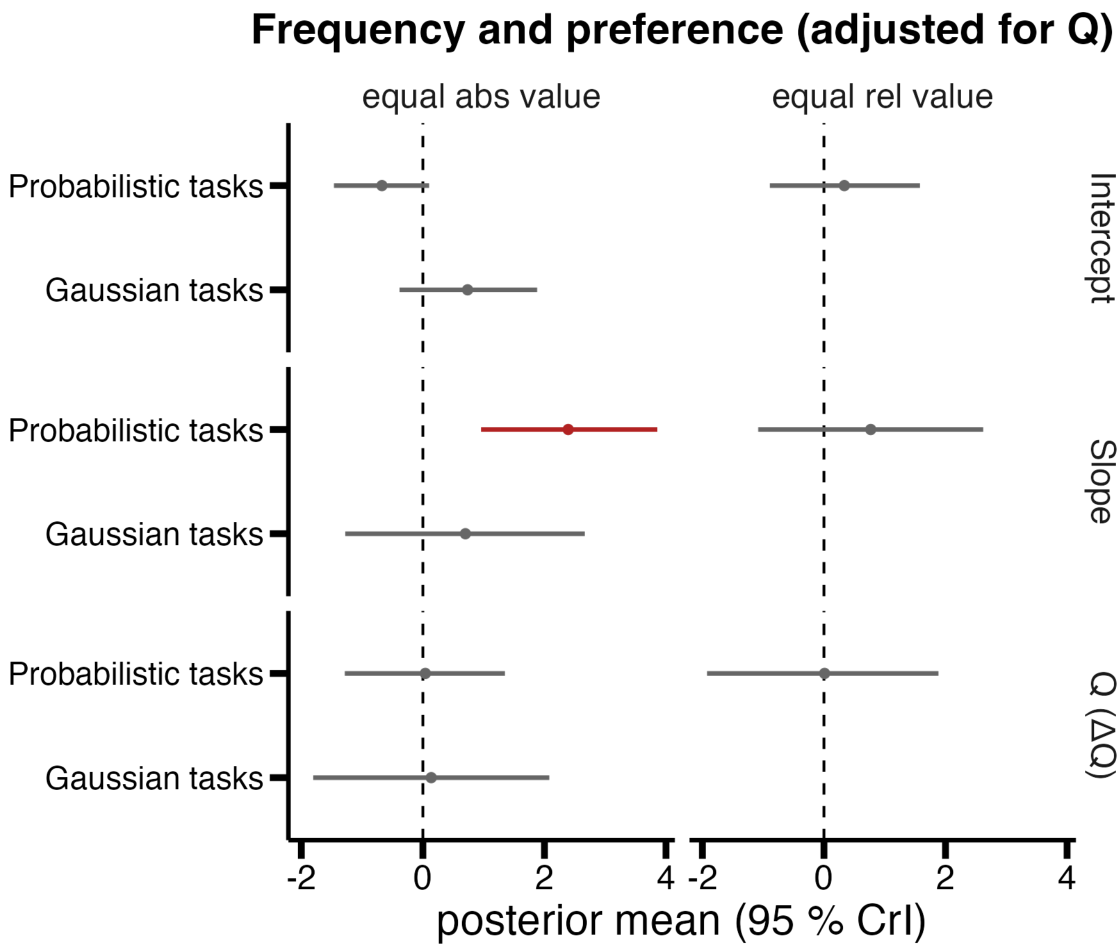

Supplementary Figure 18 Hierarchical logistic regressions. We recomputed our hierarchical logistic regressions for all of our new datasets (and both datasets from Klein et al. 2017) to predict the first transfer choice between options C (HC context/ HG context in tasks p4.1 and p4.2) and option A (LC context / LG context in tasks p4.1 and p4.2) from Q-value difference (ΔQ; C(HC) – A(LC)) and relative choice frequency (Slope) during learning (relative frequency for C in contrast to A), with random intercepts and random slopes for both predictors (ΔQ and relative choice frequency) by participant. Panels (columns) separate datasets into equal absolute value (probabilistic tasks [p1, p2, p3, Klein et al. 2017 exp2, Klein et al. 2017 exp3]; Gaussian tasks [g1, g2]) and equal relative value (probabilistic tasks p4.1 and p4.2). Points are posterior means and bars are 95% credible intervals; the vertical dashed line corresponds to 0. Red points indicate effects whose 95% CI excludes 0. As in our main analysis in the probabilistic, equal absolute value set, the frequency slope is positive and credible, predicting transfer preference after controlling for ΔQ. In the equal-relative value set, the frequency effect is numerically positive but not credible. In the Gaussian, equal absolute set, the frequency effect is likewise numerically positive but not significant. Across all analyses, ΔQ (HC − LC) estimates are centered near zero with wide CIs, indicating that adding Q-value differences at the end of the learning phase estimated via ABS or REL models do not account for transfer preference.

References

1. Molinaro, G. & Collins, A. G. E. Intrinsic rewards explain context-sensitive valuation in reinforcement learning. *PLOS Biology* **21,** e3002201; 10.1371/journal.pbio.3002201 (2023).
2. Klein, T. A., Ullsperger, M. & Jocham, G. Learning relative values in the striatum induces violations of normative decision making. *Nature Communications* **8,** 16033; 10.1038/ncomms16033 (2017).
3. Louie, K., Grattan, L. E. & Glimcher, P. W. Reward value-based gain control: divisive normalization in parietal cortex. *J. Neurosci.* **31,** 10627–10639; 10.1523/JNEUROSCI.1237-11.2011 (2011).
4. Louie, K. & Glimcher, P. W. Efficient coding and the neural representation of value. *Annals of the New York Academy of Sciences* **1251,** 13–32; 10.1111/j.1749-6632.2012.06496.x (2012).
5. Louie, K., Khaw, M. W. & Glimcher, P. W. Normalization is a general neural mechanism for context-dependent decision making. *Proceedings of the National Academy of Sciences* **110,** 6139–6144; 10.1073/pnas.1217854110 (2013).
6. Bavard, S. & Palminteri, S. The functional form of value normalization in human reinforcement learning. *eLife* **12**; 10.7554/eLife.83891 (2023).
7. Spiegelhalter, D. J., Best, N. G., Carlin, B. P. & Linde, A. The Deviance Information Criterion: 12 Years on. *J. R. Stat. Soc. Ser. B. Stat. Methodol.* **76,** 485–493; 10.1111/rssb.12062 (2014).
8. Vehtari, A., Gelman, A. & Gabry, J. Practical Bayesian model evaluation using leave-one-out cross-validation and WAIC. *Stat Comput* **27,** 1413–1432; 10.1007/s11222-016-9696-4 (2017).
9. Bavard, S., Lebreton, M., Khamassi, M., Coricelli, G. & Palminteri, S. Reference-point centering and range-adaptation enhance human reinforcement learning at the cost of irrational preferences. *Nat Commun* **9,** 4503; 10.1038/s41467-018-06781-2 (2018).
10. Sutton, R. S. & Barto, A. G. *Reinforcement Learning, second edition. An Introduction* (MIT Press, 2018).
