# Supplementary material for: Action repetition biases choice in context-dependent decision-making
Source: Commun Psychol. 2025 Nov 26;3:177. doi: 10.1038/s44271-025-00363-x (PMC12660811; doi:10.1038/s44271-025-00363-x)
Supplement: Supplementary file 3 — reporting_summary [file 44271_2025_363_MOESM3_ESM.pdf]

nature portfolio

Corresponding author(s): Ben Jonathan Wagner
bwagner@tuebingen.mpg.de

Last updated by author(s): 23.10.2025

Reporting Summary

Nature Portfolio wishes to improve the reproducibility of the work that we publish. This form provides structure for consistency and transparency in reporting. For further information on Nature Portfolio policies, see our Editorial Policies and the Editorial Policy Checklist.

Statistics

For all statistical analyses, confirm that the following items are present in the figure legend, table legend, main text, or Methods section.

n/a	Confirmed	
☐	☒	The exact sample size (*n*) for each experimental group/condition, given as a discrete number and unit of measurement
☐	☒	A statement on whether measurements were taken from distinct samples or whether the same sample was measured repeatedly
☐	☒	The statistical test(s) used AND whether they are one- or two-sided<br>*Only common tests should be described solely by name; describe more complex techniques in the Methods section.*
☐	☒	A description of all covariates tested
☐	☒	A description of any assumptions or corrections, such as tests of normality and adjustment for multiple comparisons
☐	☒	A full description of the statistical parameters including central tendency (e.g. means) or other basic estimates (e.g. regression coefficient) AND variation (e.g. standard deviation) or associated estimates of uncertainty (e.g. confidence intervals)
☐	☒	For null hypothesis testing, the test statistic (e.g. *F*, *t*, *r*) with confidence intervals, effect sizes, degrees of freedom and *P* value noted<br>*Give P values as exact values whenever suitable.*
☐	☒	For Bayesian analysis, information on the choice of priors and Markov chain Monte Carlo settings
☐	☒	For hierarchical and complex designs, identification of the appropriate level for tests and full reporting of outcomes
☐	☒	Estimates of effect sizes (e.g. Cohen's *d*, Pearson's *r*), indicating how they were calculated

Our web collection on statistics for biologists contains articles on many of the points above.

Software and code

Policy information about availability of computer code

Data collection	Participants were recruited via the online platform Prolific (www.prolific.com) and behavioral tests were conducted on the platform pavlovia.org. Further informed consent was provided via REDcap.
Data analysis	Data analysis was conducted in R using RStudio (2023.12.1). Frequentist analyses (e.g. two-sided exact binomial tests), descriptive statistics and Bayesian regressions and correlations were performed in Rstudio and standard packages (e.g. tidyverse, brms). Hierarchical Bayesian models were estimated in JAGS (Plummer, 2003) interfaced from R (via R2jags), using multiple MCMC chains with standard convergence diagnostics ($\hat{R}$, effective sample size, inspection of trace plots). Model comparison relied on WAIC (Vehtari et al. 2017).

For manuscripts utilizing custom algorithms or software that are central to the research but not yet described in published literature, software must be made available to editors and reviewers. We strongly encourage code deposition in a community repository (e.g. GitHub). See the Nature Portfolio guidelines for submitting code & software for further information.

Data

Policy information about availability of data

All manuscripts must include a data availability statement. This statement should provide the following information, where applicable:

- Accession codes, unique identifiers, or web links for publicly available datasets
- A description of any restrictions on data availability
- For clinical datasets or third party data, please ensure that the statement adheres to our policy

All newly collected datasets are available on OSF https://osf.io/zj95m/

Research involving human participants, their data, or biological material

Policy information about studies with human participants or human data. See also policy information about sex, gender (identity/presentation), and sexual orientation and race, ethnicity and racism.

Reporting on sex and gender	We report gender reported by participants in the study (m = 175, w= 176)
Reporting on race, ethnicity, or other socially relevant groupings	No data on race and ethnicity was collected.
Population characteristics	Participants were recruited via the online platform Prolific (www.prolific.com), selecting a balanced sample across the population. Inclusion criteria were fluent English and age with the range of 18 to 49 years
Recruitment	*Describe how participants were recruited. Outline any potential self-selection bias or other biases that may be present and how these are likely to impact results.*
Ethics oversight	*Identify the organization(s) that approved the study protocol.*

Note that full information on the approval of the study protocol must also be provided in the manuscript.

Field-specific reporting

Please select the one below that is the best fit for your research. If you are not sure, read the appropriate sections before making your selection.

☒ Life sciences ☐ Behavioural & social sciences ☐ Ecological, evolutionary & environmental sciences

For a reference copy of the document with all sections, see nature.com/documents/nr-reporting-summary-flat.pdf

Life sciences study design

All studies must disclose on these points even when the disclosure is negative.

Sample size	We did not run a formal prior power analysis. Instead, we decided on a minimum sample size by benchmarking against the two most closely-related studies available when we designed the work: Klein et al. (2017) and Bavard et al. (2018) reported robust effects of their normalization models in comparison to standard RL with N = 21–40 participants per experiment.
Data exclusions	We excluded 5 (out of 356) participants from analysis due to missing data or unusually long waiting periods between the learning and transfer phase (above 10 minutes).
Replication	Results can be replicated via the provided data and model code
Randomization	Not applicable. All computational analyses were performed on all participants. Participants were not assigned to groups or treatment conditions.
Blinding	not applicable

Reporting for specific materials, systems and methods

We require information from authors about some types of materials, experimental systems and methods used in many studies. Here, indicate whether each material, system or method listed is relevant to your study. If you are not sure if a list item applies to your research, read the appropriate section before selecting a response.

Materials & experimental systems

n/a	Involved in the study
☒	☐ Antibodies
☒	☐ Eukaryotic cell lines
☒	☐ Palaeontology and archaeology
☒	☐ Animals and other organisms
☒	☐ Clinical data
☒	☐ Dual use research of concern
☒	☐ Plants

Methods

n/a	Involved in the study
☒	☐ ChIP-seq
☒	☐ Flow cytometry
☒	☐ MRI-based neuroimaging

Plants

Seed stocks	*Report on the source of all seed stocks or other plant material used. If applicable, state the seed stock centre and catalogue number. If plant specimens were collected from the field, describe the collection location, date and sampling procedures.*
Novel plant genotypes	*Describe the methods by which all novel plant genotypes were produced. This includes those generated by transgenic approaches, gene editing, chemical/radiation-based mutagenesis and hybridization. For transgenic lines, describe the transformation method, the number of independent lines analyzed and the generation upon which experiments were performed. For gene-edited lines, describe the editor used, the endogenous sequence targeted for editing, the targeting guide RNA sequence (if applicable) and how the editor was applied.*
Authentication	*Describe any authentication procedures for each seed stock used or novel genotype generated. Describe any experiments used to assess the effect of a mutation and, where applicable, how potential secondary effects (e.g. second site T-DNA insertions, mosiacism, off-target gene editing) were examined.*
